# Harnessing Human Cross-Presenting CLEC9A^+^XCR1^+^ Dendritic Cells for Immunotherapy

**DOI:** 10.3389/fimmu.2014.00239

**Published:** 2014-05-22

**Authors:** Kirsteen M. Tullett, Mireille H. Lahoud, Kristen J. Radford

**Affiliations:** ^1^Mater Research Institute, University of Queensland, Brisbane, QLD, Australia; ^2^School of Medicine, University of Queensland, Brisbane, QLD, Australia; ^3^Centre for Biomedical Research, Burnet Institute, Melbourne, VIC, Australia; ^4^Department of Immunology, Monash University, Melbourne, VIC, Australia; ^5^School of Biomedical Sciences, University of Queensland, Brisbane, QLD, Australia

**Keywords:** dendritic cells, immunotherapy, cross-presentation, DC targeting, Clec9A, XCR1

## Introduction

Dendritic cells (DC) are professional antigen presenting cells (APCs) that play a pivotal role in the induction and regulation of immune responses, including the induction of cytotoxic T lymphocyte (CTL) responses. They are an important focus for the development of vaccines against cancers and many pathogens, including HIV and malaria, where CTL responses are required for protection and disease eradication. DC loaded *ex vivo* with tumor antigen (Ag) have been administered as vaccines to cancer patients for over 15 years. They are well-tolerated and induce immune responses, including some clinical regressions, but there is clearly room for improvement (1). The DC network in both mice and humans is heterogeneous, with specialized DC subsets driving specific immune functions (2). New developments in our understanding of DC biology have identified a subset of DC characterized by the expression of novel markers CLEC9A (DNGR-1) (3, 4) and XCR1 (5, 6) as being important for the induction of CTL responses (7). Vaccine strategies that deliver Ag and activators directly to CLEC9A^+^XCR1^+^ DC *in vivo* promise to overcome many of the logistical issues associated with *in vitro*-derived vaccines, allowing precision and specificity of the desired immune response (8). Here, we discuss the biological properties of CLEC9A^+^XCR1^+^ DC that make them such attractive targets for CTL vaccines and new vaccine approaches to target them *in vivo*.

## CLEC9A^+^XCR1^+^ DC are Essential for CTL Induction

The emerging complexity of the DC network and the optimal DC subset to target is the first important consideration for the design of new vaccines that target DC *in vivo*. In human and mouse, multiple DC subsets exist that vary in location, phenotype, and specialized function (2). They can be broadly classified as (i) inflammatory monocyte-derived (Mo) DC that develop from monocytes and are rapidly recruited to sites of inflammation; (ii) plasmacytoid DC (pDC) that are major producers of type I interferons (IFN) in response to TLR 7/9 ligation and are key for anti-viral immunity; and (iii) conventional DC (cDC) that can be further divided based on location into “lymphoid-resident” and “migratory” DC (2). The lymphoid-resident DC capture Ag directly in lymphoid tissues, whereas the migratory DC reside in the peripheral organs (e.g. lung, skin, and gut) where they capture Ag then migrate to lymphoid tissues to share their Ag with other lymphoid-resident DC, or present Ag directly to T cells. In both locations, cDC can be further segregated into subsets with specialized functions. Increasing evidence points to a role for the mouse CD11b^+^ cDC subset in the induction of CD4^+^ T cell responses although a similar role for the equivalent human CD1c^+^ DC subset has not yet been established (2, 9). However, it is the subset defined by expression of the C-type lectin-like receptor, CLEC9A, and the chemokine receptor, XCR1, that is crucial for the induction of CTL responses against cancers, viruses, and other pathogenic infections (2, 7).

CLEC9A^+^XCR1^+^ DC were originally identified in mice by expression of the markers CD8α on lymphoid-resident DC or CD103 on migratory DC and are commonly referred to as CD8α^+^ lymphoid and CD103^+^ migratory DC. In humans, CLEC9A^+^XCR1^+^ DC, commonly referred to as CD141^+^ DC, are found in both lymphoid and non-lymphoid tissues, including skin, gut, liver, and lungs (6, 10–13). CLEC9A and XCR1 are exclusively expressed by this unique DC subset in lymphoid and non-lymphoid tissues of both species, with the exception of low levels of expression of Clec9A by mouse pDC. As these markers combined are currently the most specific means of defining these DC in both species, we hereafter refer to them as CLEC9A^+^XCR1^+^ DC. In addition to CLEC9A and XCR1, these DC share expression of the nectin-like protein, Necl2 (14) and TLR3, and are major producers of IFN-λ after TLR3 ligation (15). Importantly, they excel at cross-presentation, the mechanism that allows exogenous Ag, such as that captured from tumors and virally infected cells to be processed and presented on MHC I for recognition by CTLs (16).

## What Makes CLEC9A^+^XCR1^+^ DC so Effective at CTL Priming?

Although other cell types, including macrophages, B cells, and other DC subsets, can cross-present under particular circumstances *in vitro* (17–20), there is substantial evidence to demonstrate that CLEC9A^+^XCR1^+^ DC are inherently more efficient at this process *in vitro* and *in vivo* (6, 7, 10, 11, 16). The precise molecular mechanisms are not understood but extensive efforts have yet to reveal specialized cross-presentation machinery unique to CLEC9A^+^XCR1^+^ DC (16). However, there are several features of these DC that collectively explain their superior cross-priming ability despite a similar Ag uptake capacity compared with other DC subsets. Firstly, CLEC9A^+^XCR1^+^ DC maintain a less acidic pH in endosomes and phagosomes, favoring cross-presentation from early endocytic vesicles (21), and facilitating cross-presentation of Ag targeted to late endosomes/lysosomes (20, 22). Secondly, CLEC9A^+^XCR1^+^ DC are more efficient at translocation of Ag from endosomes/phagosomes into the cytosol for access to the classical MHC I processing pathway (23). Thirdly, CLEC9A, a receptor for actin filaments exposed on dead cells, plays a key role in delivering Ag captured from dead cell for cross-priming (24–27). Fourthly, CLEC9A^+^XCR1^+^ DC express high levels of TLR3, a known enhancer of cross-priming (28). Finally, constitutive activation of unfolded-protein-response sensor, IRE-1α, and the transcription factor XBP-1 was recently shown to regulate cross-presentation specifically by CLEC9A^+^XCR1^+^ DC (29). There is also evidence that XCR1 and Necl2 are involved in CTL activation, although not directly via augmenting the cross-presentation pathway (5, 6, 14). These features provide a strong rationale to develop technologies that specifically deliver Ag to the cross-presentation pathway of CLEC9A^+^XCR1^+^DC *in vivo*.

## Targeting CLEC9A^+^XCR1^+^DC *in vivo*

Antibodies (Ab) specific for DC surface receptors, particularly Ag uptake receptors, can be harnessed to deliver Ag directly to DC *in vivo* (30). The choice of receptor depends on its specificity for the DC subset to be targeted in addition to the Ag processing and presentation pathway used by the receptor following internalization. A variety of C-type lectin receptors (CLR) have been exploited for this purpose, and this is reviewed elsewhere (1, 30) but for delivering Ag to CLEC9A^+^XCR1^+^ DC in mice, DEC-205 has been a major focus. Delivery of Ag via DEC-205 Ab induces both CD4^+^ and CD8^+^ T cell responses in the presence of adjuvant and is superior to *ex vivo* loaded DC vaccines at preventing tumor growth [reviewed elsewhere (31)]. Phase I/II clinical trials targeting NY-ESO-1 Ag for treatment of multiple solid malignancies expressing this Ag are in progress utilizing CDX-1401, a fully humanized Ab against DEC-205 (CellDex Therapeutics). In humans, DEC-205 is widely expressed on all DC, in addition to B cells, T cells, and NK cells. Although CLEC9A^+^XCR1^+^ DC, CD1c^+^ DC, pDC, and MoDC have been shown to process and present Ag delivered by DEC-205 to CD4^+^ and CD8^+^ T cells *in vitro* (20, 31–33), limited direct comparisons suggest CLEC9A^+^ XCR1^+^ DC to be more effective at cross-presentation (20). This is likely due to the preferential trafficking of DEC-205 to late endosomes, which typically favors Ag processing via the MHC II pathway (34), whilst still allowing cross-presentation by CLEC9A^+^XCR1^+^ DC (20).

An attractive approach is to more specifically deliver Ag to CLEC9A^+^XCR1^+^ DC using Ab or ligands specific for CLEC9A (3, 4) or XCR1 (35). Studies utilizing Clec9A for Ag delivery in mice observe effective CD8^+^ T cell responses and, surprisingly, superior CD4^+^ T cell immunity when directly compared to DEC-205, even in the absence of adjuvant (3, 4, 36). Key reasons for the efficacy of targeting Clec9A include its intracellular trafficking, as Clec9A delivers Ag to early and recycling endosomes (27), and the persistence of anti-Clec9A Ab in serum, resulting in prolonged Ag presentation (36). Determining the molecular interactions of CLEC9A following internalization and how this influences Ag trafficking and processing, will undoubtedly shed light on the basis for Clec9A targeting efficacy.

Anti-human CLEC9A Ab can deliver Ag to human CLEC9A^+^XCR1^+^ DC for processing and presentation to both CD4^+^ and CD8^+^ T cell lines *in vitro* (37). This provides proof-of-principle and a strong rationale to further develop anti-human CLEC9A Ab for vaccines and more comprehensively compare with DEC-205 Ab and other approaches that target multiple DC subsets. Such studies have been limited due to difficulties in obtaining sufficient numbers of human CLEC9A^+^XCR1^+^ DC for detailed functional analysis, but are now feasible with the development of new humanized mouse models, where functional human CLEC9A^+^XCR1^+^ DC develop and can be targeted with CLEC9A or DEC-205 Abs *in vivo* (38).

## Adjuvants for Activation of CLEC9A^+^XCR1^+^DC

Early DC clinical trials and mouse studies investigating Clec9A or DEC-205 targeting Ab have clearly demonstrated a requirement for DC activation in order to induce optimal CTL responses (31, 39). TLR ligands are some of the most promising adjuvants currently being evaluated in the clinic and differential expression of TLR by DC subsets could profoundly affect the choice of adjuvant. This is a particularly important consideration for the preclinical evaluation of vaccines targeting CLEC9A^+^XCR1^+^ DC since TLR expression varies in mouse and human DC subsets. The TLR9 ligand, CpG, has been widely used as an adjuvant in mice, including with Clec9A Ab (36) and has been evaluated clinically, with limited adverse effects, as an adjuvant in cancer chemotherapy and *ex vivo* DC vaccines (40). Whilst TLR9 is widely expressed in mice, including by CLEC9A^+^XCR1^+^ DC, in humans it is restricted to pDCs (39). However, activation of human pDC by CpG induces large amounts of type I IFN that could potentially play an important bystander function for activation of CLEC9A^+^XCR1^+^ DC and subsequent induction of anti-tumor responses (41, 42). In contrast to their mouse counterparts, human CLEC9A^+^XCR1^+^ DC also lack expression of TLR4 but express TLR8, which is not functional in mice (39).

A TLR7/8 ligand, R848 or resiquimod, has been FDA approved for topical use and is currently undergoing clinical trials with DEC-205 (CDX-1401, CellDex) (43). It also activates CD1c^+^ DC via TLR8 and pDC via TLR7. Its potential to be used in vaccines remains to be determined, with murine studies indicating that its short half-life and formulation may not be ideal for activating DC locally to initiate adaptive immune responses, and it has been implicated in severe side effects observed in clinical trials (43).

The TLR3 ligand, polyI:C, is emerging as an attractive adjuvant to combine with DC targeting Ab, as TLR3 expression is conserved across human and mouse CLEC9A^+^XCR1^+^ DC. PolyI:C was found to be the optimal adjuvant to use in combination with DEC-205 targeting Ab in mice (44). The poly I:C derivatives Hiltonol and Ampligen are well-tolerated in humans and induce a type I IFN response mimicking that of a viral infection (45). These are now being evaluated in clinical trials in conjunction with DEC-205 targeting Ab (CellDex Therapeutics; NCT00948961).

## Conclusion

There remains a great need for the development of vaccines that elicit effective anti-viral and anti-tumor CTL responses. The discovery of the CLEC9A^+^XCR1^+^DC in mice and humans, as a subset specialized for Ag cross-presentation and cross-priming CTL, has revealed promising new avenues for vaccine design. Yet, the contribution of other DC subsets to the efficacy of this process is still to be determined. Thus, the questions remain: is it more effective to deliver Ag to the CLEC9A^+^XCR1^+^DC that are best-equipped for cross-presentation, or will co-delivery to other DC subsets provide help? Which receptors will best deliver the Ag to the required intracellular compartments, and which adjuvants will best enhance immune responses? Studies to date suggest that targeting CLEC9A^+^XCR1^+^ DC *in vivo*, together with adjuvants to specifically activate these DC, offers great promise. The advancement of humanized mouse models allowing for development of CLEC9A^+^XCR1^+^ DC and other DC subsets, will enable these and other questions to be answered, and facilitate translation from bench to bed-side.

## Conflict of Interest Statement

Mireille H. Lahoud and Kirsteen M. Tullett are listed as inventors on patent applications relating to Clec9A. Kristen J. Radford has no conflicts of interest to report.
